# Glossary

**Published:** 2006

Acetaldehyde: A toxic byproduct of alcohol *metabolism*.
Acetate: A salt or ester of acetic acid; produced from the *metabolism* of *acetaldehyde*.
Adduct: Product of the addition of one compound (e.g., *acetaldehyde)* to another compound (e.g., DNA).
Adenosine triphosphate (ATP): A molecule, generated largely in the *mitochondria*, that provides the energy needed for many key *metabolic* reactions.
Alcohol dehydrogenase (ADH): An *enzyme* that breaks down alcohol by *oxidation*, converting it to *acetaldehyde*. (See *cytochrome P450.)*
Aldehyde dehydrogenase (ALDH): An *enzyme* that converts *acetaldehyde* to *acetate*.
Allele: One of two or more variants of a certain gene.
Amine: A type of organic compound that contains nitrogen as a central atom.
Amino acids: The principal building blocks of *proteins* and *enzymes*.
Amino group: A group of atoms found in all *amines* and *amino acids*.
Antibody: A *protein* produced by certain immune cells that recognizes and binds to foreign proteins, leading to the destruction of those proteins.
Antibody-dependent cell-mediated cytotoxicity (ADCC): A mechanism of cell-mediated immunity whereby certain immune cells actively break up a target cell that has been bound by specific *antibodies*.
Antioxidant: A substance, such as glutathione, vitamin E, or an *enzyme*, that inhibits *oxidation* and that scavenges *free radicals* and protects the cell against damage caused by these radicals.
Apoptosis: Cell death in which the affected cell participates by activating a cascade of biochemical reactions that lead to death; also known as programmed cell death or cell suicide.
Aromatic amino acids: A class of *amino acids*, including phenylalanine and tryptophan, in which some of the constituent atoms form a ring.
Catalase: An e*nzyme* that catalyzes the decomposition of hydrogen peroxide into water and oxygen.
Central vein: Blood vessel located in the center of each liver *lobule* through which cleansed blood exits the lobule and which feeds into the hepatic vein; also called hepatic venule.
Cytochrome P450: A family of *cytochromes*, one of which (CYP2E1) can oxidize alcohol to form *acetaldehyde*; high alcohol levels stimulate CYP2E1 activity.
Cytochromes: Specialized *enzymes* within *mitochondria* and other cell structures. Different cytochromes play important roles in metabolizing toxic substances, drugs, and other chemicals, as well as in producing *adenosine triphosphate (ATP)*.
Cytokines: A family of molecules, produced primarily by cells of the immune system, that regulate cellular interactions and other functions. Many cytokines play important roles in initiating and regulating inflammation.
Cytoplasm: The substance filling the cell, including the *cytosol* as well as *mitochondria*, *endoplasmic reticulum*, and other cell structures (organelles) but excluding the nucleus.
Cytosol: The fluid portion of the *cytoplasm*.
Electron: A subatomic particle with a negative charge.
Endocytosis: Mechanism by which specific molecules are ingested into the cell.
Endoplasmic reticulum: A system of folded membranes that loop back and forth, spreading throughout the *cytoplasm* and providing a large surface area for cell reactions.
Endothelial cells: Type of cell lining the body cavities and blood vessels; control the passage of materials and the transit of white blood cells into and out of the bloodstream.
Enzyme: A substance, usually a *protein*, that directs and accelerates chemical reactions in the body but does not itself undergo permanent change.
Expression (i.e., gene expression): The process by which the genetic information encoded in a gene’s DNA sequence is converted into a functional *protein*.
Fatty Acids: A major component of fats that is used by the body for energy and tissue development.
Fibrosis: The formation of scar tissue.
Free radicals: Highly reactive molecular fragments that frequently contain oxygen. (See *reactive oxygen species [ROS]*.)
Genotype: The complete genetic makeup of an organism determined by the particular combination of *alleles* for all genes.
Hepatic vein: A large vessel that receives blood after it has passed through the *central veins* of the liver *lobules*.
Hepatocytes: The principal cells of the liver, which carry out most of the liver’s metabolic activities.
Heterozygous: Carrying two different *alleles* of a given gene.
Homozygous: Carrying two copies of the same *allele* of a given gene.
Hyperlipidemia: Excess fat in the blood.
Hyperuricemia: Excess uric acid in the blood.
Hypoxia: Lower-than-normal levels of oxygen.
Isozymes/Isoenzymes: *Enzymes* that differ in *amino acid* sequence but catalyze the same chemical reaction.
Ketosis: Abnormal accumulation in the body of ketones, which are end products of fatty acid metabolism. Ketosis occurs when the body cannot metabolize sufficient carbohydrates to generate the energy needed (e.g., in patients with diabetes or during starvation).
*K_m_*: A measurement used to describe the activity of an *enzyme*. It describes the concentration of the *enzyme’s substrate* at which the *enzyme* works at 50 percent capacity.
Kupffer cells: Specialized immune cells in the liver that filter bacteria and other foreign substances from the blood and produce antibodies and *cytokines*. (See also *sinusoids*.)
Lactic acidosis: A condition characterized by the accumulation of lactic acid in bodily tissues.
Lipids: Fatty substances, including simple fats, their major components (i.e., *fatty acids*), and various fat-soluble substances (e.g., cholesterol).
Lipid peroxidation: The sequential breakdown of fatty substances in cells by chemical *oxidation*, leading eventually to the destruction of membranes within and surrounding the cell.
Lobule: A cylindrical structure about 2 millimeters in diameter that is the basic functional unit of the liver. The liver can be composed of up to 100,000 lobules.
Macrophage: A type of immune cell that ingests foreign particles and microorganisms and synthesizes *proteins* and other substances important in inflammatory responses, including *cytokines*. Macrophages that reside in the liver are called *Kupffer cells*.
Metabolism: The totality of chemical reactions occurring in a cell, an organ, or the body. The term sometimes is applied more narrowly to the breakdown of a particular substance (e.g., alcohol) by specific *enzymes*.
Microsomes: Small *vesicles* derived from fragmented *endoplasmic reticulum* produced when tissues such as liver are mechanically broken (homogenized). Microsomes contain the cell’s *cytochrome P450 (CYP) enzymes*, involved in *oxidative metabolism*.
Microsomal ethanol-oxidizing system: An *enzyme* system involving *cytochrome P450* that breaks down alcohol and generates toxic products, such as *acetaldehyde* and *reactive oxygen species (ROS)*.
Microtubules: Any of the minute tubules in cell *cytoplasm* that are composed of the *protein* tubulin and form important structural components.
Mitochondria: Structures within cells that generate most of the cells’ energy through the production of *adenosine triphosphate (ATP)*.
Mitochondrial electron transport system: see *Respiratory chain*.
NAD/NADH: Nicotinamide adenine dinucleotide (NAD) is a molecule that binds with hydrogen atoms and becomes reduced NAD, or NADH, during alcohol *metabolism* and other chemical reactions in the cell. NAD and NADH move hydrogen atoms back and forth between various *oxidation–reduction* reactions, helping to maintain balance between *oxidation* and *reduction* in the cell.
Oxidation: A chemical reaction that results in a loss of *electrons* by a substance and which usually involves removing a hydrogen atom from a molecule or adding oxygen to it, or both. (See *reduction*.)
Oxidative stress: An imbalance between oxidants (e.g., *free radicals*) and *antioxidants* that can lead to excessive *oxidation* and cell damage.
Perivenous: Referring to the region of a liver *lobule* surrounding the *central vein*.
Peroxisomes: A cytoplasmic cell organelle containing *enzymes* that act in the production and decomposition of hydrogen peroxide.
Phospholipid: A *lipid* that contains a phosphate group.
Polymorphism: Existence of a gene in several *allelic* forms.
Proliferation: The growth and reproduction of cells.
Proteins: Molecules composed of chains of *amino acids* linked together. Proteins help maintain the cell’s structure and participate in many biological functions, including the regulation of metabolic reactions. The shape and function of a protein is determined by the sequence of its *amino acids*.
Reactive oxygen species (ROS): Highly reactive oxygen-containing *free radicals* that are generated during *oxidative metabolism*. ROS can react with and damage *lipids, proteins*, and DNA in cells, causing *oxidative stress*. Common ROS include hydrogen peroxide, *superoxide* radicals, and hydroxyl radicals.
Receptor: A *protein* on the surface of a cell that recognizes and binds to chemical messengers.
Redox/Redox state: Shorthand for *reduction/oxidation* reactions. The term redox state is often used to describe the balance of *NAD* and *NADH* in a biological system such as a cell or organ. An abnormal redox state can develop in a variety of deleterious situations.
Reduction: The reverse of *oxidation*, reduction is a chemical reaction that results in a gain of *electrons* by a substance and which usually involves removing an oxygen atom from a molecule, or adding hydrogen to it, or both.
Respiratory chain: The *electron* transport system located in the *mitochondria*, in which *electrons* released by *NADH* are passed on to a series of other molecules that first accept the *electrons* and then pass them on to the next molecule in the chain. The *electrons* ultimately are transferred to oxygen to generate water. These successive reactions provide enough energy to drive the synthesis of *adenosine triphosphate* (*ATP)* molecules.
Retinol: Vitamin A.
Sinusoids: Channels in a liver *lobule* that bring blood and nutrients to the *hepatocytes*, similar to capillaries in other organs. Sinusoids are lined with *endothelial cells* and *Kupffer cells*.
Stellate cell: A star-shaped liver cell that serves as the primary storage site for vitamin A compounds and fat molecules; activation of stellate cells plays a central role in the development of *fibrosis*.
Substrate: A substance upon which an *enzyme* acts.
Superoxide: A destructive *reactive oxygen species (ROS)* produced as a byproduct of some *oxidation* reactions.
Tumor necrosis factor alpha (TNF-α): A type of *cytokine* that promotes inflammatory responses, stimulates neutrophils and *macrophages*, induces fever, and induces *macrophages* to produce *cytokines*.

